# Micrometer-resolution X-ray tomographic full-volume reconstruction of an intact post-mortem juvenile rat lung

**DOI:** 10.1007/s00418-020-01868-8

**Published:** 2020-03-18

**Authors:** Elena Borisova, Goran Lovric, Arttu Miettinen, Luca Fardin, Sam Bayat, Anders Larsson, Marco Stampanoni, Johannes C. Schittny, Christian M. Schlepütz

**Affiliations:** 1 Swiss Light Source, Paul Scherrer Institute, 5232 Villigen PSI, Switzerland; 2 Institute of Anatomy, University of Bern, 3012 Bern, Switzerland; 3 Center for Biomedical Imaging, École Polytechnique Fédérale de Lausanne, 1015 Lausanne, Switzerland; 4 Institute for Biomedical Engineering, ETH Zurich, 8092 Zurich, Switzerland; 5 European Synchrotron Radiation Facility, 38043 Grenoble, France; 6 Department of Pulmonology and Clinical Physiology, Grenoble University Hospital, 38700 Grenoble, France; 7 Inserm UA7, Synchrotron Radiation for Biomedicine Laboratory (STROBE), University Grenoble Alpes, 38000 Grenoble, France; 8 Department of Surgical Sciences, Uppsala University, 75185 Uppsala, Sweden

**Keywords:** X-ray tomography, Fast tomography, Image reconstruction, Large volume tomography, Lung imaging

## Abstract

In this article, we present an X-ray tomographic imaging method that is well suited for pulmonary disease studies in animal models to resolve the full pathway from gas intake to gas exchange. Current state-of-the-art synchrotron-based tomographic phase-contrast imaging methods allow for three-dimensional microscopic imaging data to be acquired non-destructively in scan times of the order of seconds with good soft tissue contrast. However, when studying multi-scale hierarchically structured objects, such as the mammalian lung, the overall sample size typically exceeds the field of view illuminated by the X-rays in a single scan and the necessity for achieving a high spatial resolution conflicts with the need to image the whole sample. Several image stitching and calibration techniques to achieve extended high-resolution fields of view have been reported, but those approaches tend to fail when imaging non-stable samples, thus precluding tomographic measurements of large biological samples, which are prone to degradation and motion during extended scan times. In this work, we demonstrate a full-volume three-dimensional reconstruction of an intact rat lung under immediate post-mortem conditions and at an isotropic voxel size of (2.75 µm)^3^. We present the methodology for collecting multiple local tomographies with 360° extended field of view scans followed by locally non-rigid volumetric stitching. Applied to the lung, it allows to resolve the entire pulmonary structure from the trachea down to the parenchyma in a single dataset. The complete dataset is available online (https://doi.org/10.16907/7eb141d3-11f1-47a6-9d0e-76f8832ed1b2).

**Electronic supplementary material:**

The online version of this article (10.1007/s00418-020-01868-8) contains supplementary material, which is available to authorized users.

## Introduction

In the field of X-ray tomographic imaging, third-generation synchrotron X-ray sources have established themselves as invaluable experimental facilities to obtain the three-dimensional (3D) information on internal structure with unprecedented spatial and temporal resolutions (Eriksson et al. 2014; Stock 2008a). The possibility to generate 3D density maps of small samples down to micrometer resolution (Flannery et al. 1987) together with the access to different contrast mechanisms (absorption, phase, scattering) has led to a number of different available phase-contrast imaging (PCI) techniques (Bravin et al. 2013; Nugent 2010; Wilkins et al. 2014) and hence enabled the study of structural properties of a wide range of materials on different length and time scales, from millimeters down to tens of nanometers (Villanova et al. 2017). With the most recent advances in detector technology and experimental setup design, it is possible to routinely acquire a tomographic scan in 1 s (Lovric et al. 2016), with the fastest measurements reaching up to 20 (dos Santos Rolo et al. 2014; Maire et al. 2016) and even 208 (García-Moreno et al. 2019) full tomographic scans per second.

In spite of these advances, a typical limitation is the size of the achievable field of view. Depending on optical magnification and detector pixel dimensions, only millimeter- to centimeter-sized objects can be imaged with a single acquisition. Various approaches have been previously developed to enable high-resolution tomography of extended objects (Kyrieleis et al. 2009). The common idea is to image parts of the sample and merge them either before or after the tomographic reconstruction. Independent of the method, the process is time- and resource-consuming, and although more efficient techniques have been developed (Du et al. 2018; Vescovi et al. 2018) studies of large extended objects have been limited to static samples so far.

X-ray tomography has proven to be a powerful method to study the complex multi-scale architecture of lung, consisting of large airways, which end in thousands to millions of tiny structures, alveoli, tightly packed in a small space (Weibel 2009). Although a lot of progress has been made in understanding lung micro-mechanics, lung diseases still stay one of the major health burdens worldwide (Ferkol and Schraufnagel 2014), and, therefore, a better understanding of lung structure and function from a large scale down to the micrometer level remains essential.

Initial studies of lung micro-mechanics have primarily used a well-established method of sectioning histology combined with a follow-up optical or electron microscopy, which provided a better understanding of the role that the microstructure as well as the whole lung volume plays in the physiology of breathing and gas exchange processes (Mercer et al. 1994; Yager et al. 1994). This method can be also used to obtain 3D datasets (Grothausmann et al. 2017). However, it suffers from some obvious drawbacks and limitations: chemical and mechanical tissue damage is unavoidable during the preparation stage and the whole process is very time-consuming. Furthermore, distortions of the histological sections make the alignment of the sections in the 3D dataset a tedious task and cause many errors and artefacts.

Much progress has been made in X-ray computed tomography of lung, which currently allows not only non-destructive (Haberthür et al. 2013) but also non-embedded imaging of lungs thanks to PCI methods and technical advances at synchrotron beamlines (Lovric et al. 2013; Sera et al. 2013). Tsuda et al. (2008) pointed out that an approach combining high-resolution 3D imaging with computer modeling provides an extremely powerful tool to assess various features of the lung. Indeed, in recent studies this method appeared more often (Burrowes et al. 2017). For example, Broche et al. (2017); Perlman and Wu (2014); Rausch et al. (2011) applied it to X-ray tomographic data to investigate alveolar overstretching and stress during mechanical lung ventilation; Berg and Robinson (2011); Suki and Parameswaran (2015) studied destruction processes caused by emphysema progressive disease; Burrowes et al. (2014) showed application of modeling techniques to better understand obstructive lung diseases; Hwang et al. (2013); Kumar et al. (2013) performed morphological lung characterization.

All the studies mentioned above require adequately resolved data at the spatial scale of 2 µm and less (Xiao et al. 2013), which is until now not easy to achieve on non-fixed tissue. A general challenge in high-resolution tomography of soft tissues is that samples are degrading rapidly, leading to movements and, consequently, to low image quality. Minimization of the total duration of the experiment plays a crucial role in avoiding these issues. Additionally, in case of the lung tissue, images must be acquired no longer than 20–30 min post-mortem to preserve quasi in vivo conditions (Lovric et al. 2017a; Oikonomidis et al. 2017b). For comparison, in Sera et al. (2013) recording one tomographic volume with 2.6 µm pixel size took about 10 min. Doing any sort of mosaic to cover the full lung volume scales this number up to hours of radiation exposure. To stay within the time limit, usually either the resolution or the field of view has to be sacrificed. As an example, in the recent work by Murrie et al. (2015) the full lung at pixel sizes of 10.6 µm and 12.9 µm was acquired in 16.7 min.

In this study, we present a methodology for fast large-volume high-resolution X-ray tomography applied to imaging of a full rat lung within the 20–30 min time limit dictated by the effects of tissue degradation. The whole sample volume was covered by a contiguous 3D mosaic of so-called wide-field scans (Haberthür et al. 2010; Kyrieleis et al. 2009; Stock 2008b), which allowed to almost double the field of view of a single acquisition. Sufficient overlap was chosen between the individual scans to stitch them together after the reconstruction.

This classical mosaic method of scanning large samples is also known as the local tomography approach. Apart from being time- and dose-inefficient compared to recently developed methods (Du et al. 2018), it has one key advantage. In a slowly degrading and moving sample, each tomographic acquisition is short enough to act as a snapshot of the sample in a quasi-steady state. Each reconstructed volume is therefore free from any motion artefacts and contains reliable structural information. Clearly, as different neighboring scans are recorded at different points in time, their overlapping regions suffer from structural changes that have to be taken into account. Thanks to a recently developed non-rigid stitching procedure NRStitcher (Miettinen et al. 2019), these deformations can be easily handled even for a large number of mosaic tiles. In contrast, in other more dose-efficient methods, parts of each projection are imaged at different times. In particular, in the Tomosaic framework (Vescovi et al. 2018), series of projections are acquired at several offset positions with respect to the fixed rotation axis in a “ring in a cylinder” manner. Before the reconstruction, either two-dimensional projections at each angular position are assembled first or a full sinogram of each slice is stitched together. For such a reconstruction to work, no motion or changes larger than the pixel size during the entire acquisition process can be tolerated. Unfortunately, this condition is usually not met by fresh biological tissue samples, as is the case for an unprepared fresh intact lung.

The paper is organized as follows. In Sect. “Materials and methods”, we provide a detailed technical description of the setup and data acquisition scheme. In Sect. “Data processing”, data reconstruction and stitching of individual volumes is described. The final dataset is presented in Sect. “Results and discussion” along with a discussion of its further possible applications.

## Materials and methods

### Animal preparation

A 13-day-old Wistar rat (strain of the Central Animal Facility of the University of Bern) was anesthetized by an intraperitoneal injection of fentanyl, midazolam, and medetomidine (0.005, 2.0, 0.15 mg/kg body weight) (Erhardt et al. 2011). After a tracheotomy, an endotracheal cannula was fixed and the animal was placed in a customized sample holder (Lovric et al. 2017a) in an upright position. Next, ventilation was started using a small animal ventilator (FlexiVent, SCIREQ Inc. Montreal, Canada) and the animal was positioned on the measurement stage. Immediately prior to imaging, an overdose of pentobarbital was administered to euthanize the animal. During the subsequent tomographic imaging, the intrapulmonary pressure was kept at 10 cm water column.

Prior to the experiment, the rat was kept in a ventilated cage with filtered air in a 12/12 h light/dark cycle. It received standard food and water ad libitum. All procedures involving animal handling and experiments were approved and supervised by the Swiss Agency for the Environment, Forest and Landscape as well as the Veterinary Service and the ethics committee for animal experimentation of the Canton of Bern and the Canton of Aargau.

### Setup description

X-ray tomographic microscopy measurements were carried out at the TOMCAT beamline X02DA of the Swiss Light Source (SLS) facility at Paul Scherrer Institute (PSI) (Stampanoni et al. 2010). The imaging protocol and acquisition parameters used in this experiment were based on previously established results by Lovric et al. (Lovric et al. 2013, 2016, 2017a). In short, the quasi-parallel X-ray beam produced by the 2.9 T superbend was monochromatized to an energy of 21 keV using a Ru/C double-multilayer monochromator. The radiation illuminated the sample, which was placed 25 m away from the source. X-rays were converted to visible light with a 150 µm thick LuAG:Ce scintillator placed 100 mm downstream of the sample to allow for some degree of propagation-based edge enhancement necessary for the phase-contrast reconstruction. The X-ray image on the scintillator was optically magnified by a factor of four using a high numerical aperture macroscope (Optique Peter, France) (Bührer et al. 2019) onto the imaging chip of the GigaFRoST high-speed imaging and readout system (Mokso et al. 2017). This setup yielded an effective pixel size of 2.75 × 2.75 µm^2^. The field of view had to be cropped in the vertical direction due to the finite vertical beam size at this X-ray energy and finally covered an area of 5.5 × 3.0 mm^2^ (horizontal × vertical).

### Data collection strategy

As discussed in the introduction, the post-mortem relaxation and motion of the global lung structure and the surrounding animal body during the time required to image the entire organ dictated the use of fast scans in a local tomography mode to avoid motion artefacts in the reconstruction of the microscopic lung structure. The basic data collection strategy was thus to acquire a 3D mosaic of tiled scan volumes covering the full extent of the lung. Individual tomographic scans corresponded to sub-regions of the lung, which needed to be stitched together during the data processing phase. To determine the local registration transformations between neighboring data sets, a sufficiently large overlap between acquired volumes needed to be taken into account during the measurements.

The second paramount requirement was that the total scan time for the entire lung structure was as short as possible and definitely shorter than the time it takes for a perceivable structural degradation to set in. Namely, we have found earlier that typically a rapidly emerging stiffness increase of the lung is observed after the immediate post-mortem state as well as a significant decrease in the thickness of the alveolar septa (walls separating the air spaces in the gas exchange area), which might be best explained by a halted blood circulation and draining of the blood into the lower half of the animal (Lovric et al. 2017a).

The overall experiment time depended on the number of individual scans, the time it took to re-position the sample between scans, and additional overheads due to data readout and storage, scan and camera initialization, etc. When devising the scanning scripts, special care was taken to minimize these overheads as much as possible, but the remaining overheads still accounted for a combined 3–4 s time interval before and after every tomographic scan. On the other hand, the overhead associated with a constant speed translation of the sample due to acceleration, deceleration, and settling times was of the order of a few 100 ms per motion event in addition to the actual translation. Thus, the most efficient way to reduce the overall overhead of the full lung measurement was to reduce as much as possible the total number of individual tomographic scans that needed to be performed and hence also the total number of sample translations.

This was achieved by recording so-called extended field of view scans, also referred to as half-object acquisition, wide-field scans, or 360-degree scans (Haberthür et al. 2010; Kyrieleis et al. 2009; Stock 2008b). Thanks to the essentially parallel beam of X-rays provided by the synchrotron source, X-ray projections over an angular range of 180° are sufficient for a standard tomography reconstruction (see also similar techniques for cone-beam CT systems in Cho et al. (1995); Wang (2002)). As a consequence, the horizontal field of view of the scan can be nearly doubled by positioning the projected location of the rotation axis near one side of the detector and by rotating the sample over 360° instead of 180°. The reconstructed scan volume for this geometry is almost four times larger in its horizontal extent than for the original 180-degree scan, thus reducing the total number of scans required to cover the full lung volume by essentially a factor of four. The price to pay is two times larger number of projections to be acquired during the scan to achieve the same sampling rate as for the standard case, thus doubling the time it takes to acquire each tomographic scan. In the present case, the doubling of the scan time still resulted in a sufficiently short scan duration to fulfill the requirement that no motion artefact should have occurred within that scan.

Between individual acquisitions, the sample needed to be re-positioned along one of the three principal axes defining the mosaic tiling blocks. Since in our setup the vertical translation axis was the fastest of the three, moving at 550 µm/s compared to the 220 µm/s for both of the horizontal translations, and the vertical displacement was smaller than the horizontal one, the vertical axis was chosen as the fastest stepping axis. The mosaic was thus acquired in successive vertical stacks of scans at different horizontal positions. Additionally, the mosaic was traversed in a zigzag fashion to always visit the nearest unmeasured volume in each motion step.

### Data acquisition

High-resolution tomographic data acquisition of the full lung started immediately after euthanizing the animal and was completed in under 22 min, which was within the 20–30 min time limit to image soft tissue before structural degrading started to take place (Lovric et al. 2017a; Oikonomidis et al. 2017b). The approximate location and extent of the full lung within the animal chest as positioned in the customized sample holder was known from previous experiments. To verify the exact boundaries for the large-volume mosaic scan protocol, a few (less than 10) radiographic snapshots with total X-ray exposure times of less than 30 ms each were acquired and the sample positioning was adjusted accordingly.

The full lung was covered by 63 individual volumes with a 3 × 3 × 7 (width × length × height) mosaic geometry. The scheme implemented in the scanning script involved several steps. First, to avoid additional translation motions, a set of calibration images (50 dark frames with the beam off and 300 flat-field frames with the beam on but without the sample) was recorded only once before the mosaic acquisition. Then, a repetitive process of tomographic scan acquisition and either vertical or horizontal linear translation started. As the sample was primarily scanned column-wise, one horizontal motion occurred after every six vertical shifts.

Information from the log files with setup parameters and time references of the recorded data, saved after each individual acquisition, allowed statistical quantification of the duration of each step:

- Wide-field tomography scan: 9.0 s (average). Each individual 360-degree scan covered a physical volume of 10.8 × 10.8 × 3.0 mm^3^ (width × length × height) and consisted of 2000 projections, each with a 2.5 ms exposure time. It was acquired during continuous rotation of the sample at an angular speed of 72°/s, resulting in an overall acquisition time of 5 s per scan. The additional 4 s are accounted for by overheads in the acquisition script and camera readout. Each dataset of radiographic projections together with their corresponding recorded angular positions was saved in a hierarchical data file format (HDF5) (De Carlo et al. 2014).
- Vertical translation: 8.0 s (average). During a single vertical translation, the sample was shifted by 2.75 mm. At the constant motor speed of 550 µm/s, this motion corresponded to 5 s with the additional 3 s required to accelerate/decelerate the motor, to adjust to the correct position in agreement with the linear encoder readback and get ready for the next acquisition.
- Horizontal translation: 35.5 s (average). After every six vertical translations and seven tomographic acquisitions, the sample was moved in the horizontal plane along one of the axes depending on whether a central or an edge region was scanned next. This motion over 7.2 mm was finished on average in 35.5 s. The larger shift distance in comparison with the vertical translation was due to a larger beam size in the horizontal direction and the wide-field acquisition mode. As with the vertical translation, an overhead of about 3 s was required to get into scan ready position in addition to the constant motor motion at 220 µm/s.

**Fig. 1:**
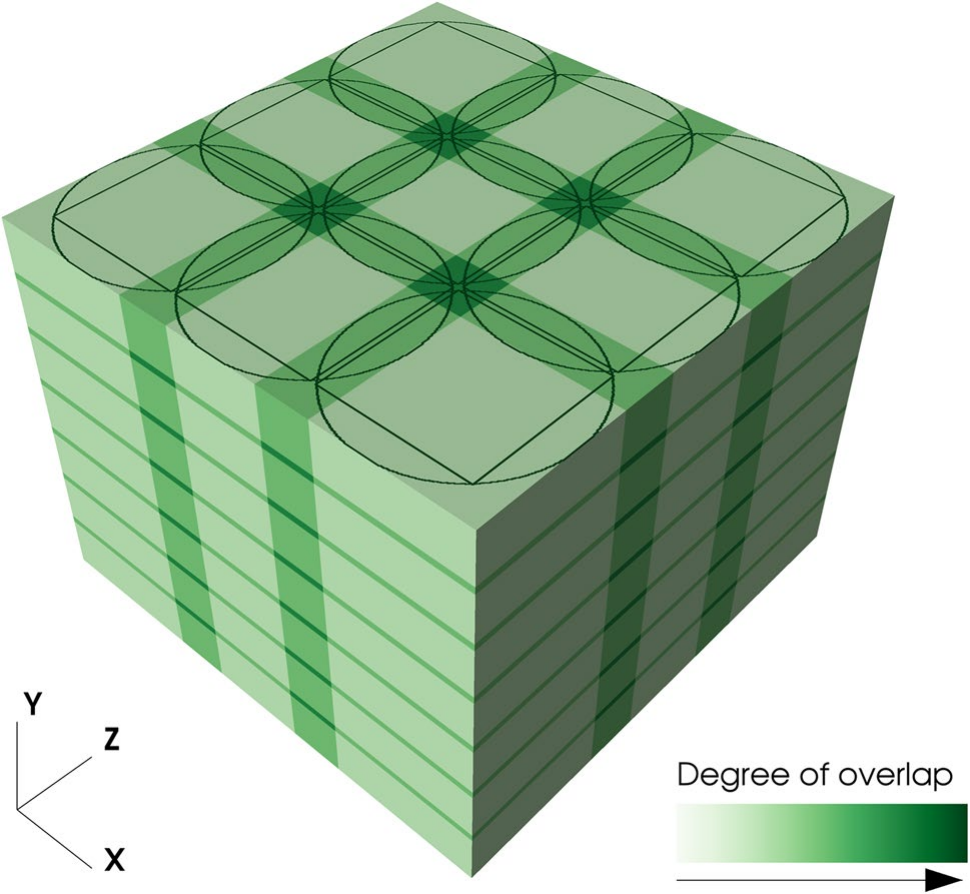
A view of the tomographic mosaic geometry with 3 × 3 × 7 tiles. Color shading highlights the overlapping areas between adjacent volumes, while the thin black lines on the top of the cube indicate the largest square and circular area which contain valid (in terms of completeness for tomographic reconstruction) data, respectively

Figure 1 presents the geometry of the final mosaic where both the full volumes as well as the valid (in terms of completeness) reconstruction regions are shown. The overlap of the inner squares of neighboring circular reconstruction regions is 100 pixels, corresponding to approximately 4% of the valid and 30% of the full surface area of a single slice. The vertical overlap between the volumes is set to 100 pixels.

## Data processing

### Tomographic reconstruction

**Fig. 2:**
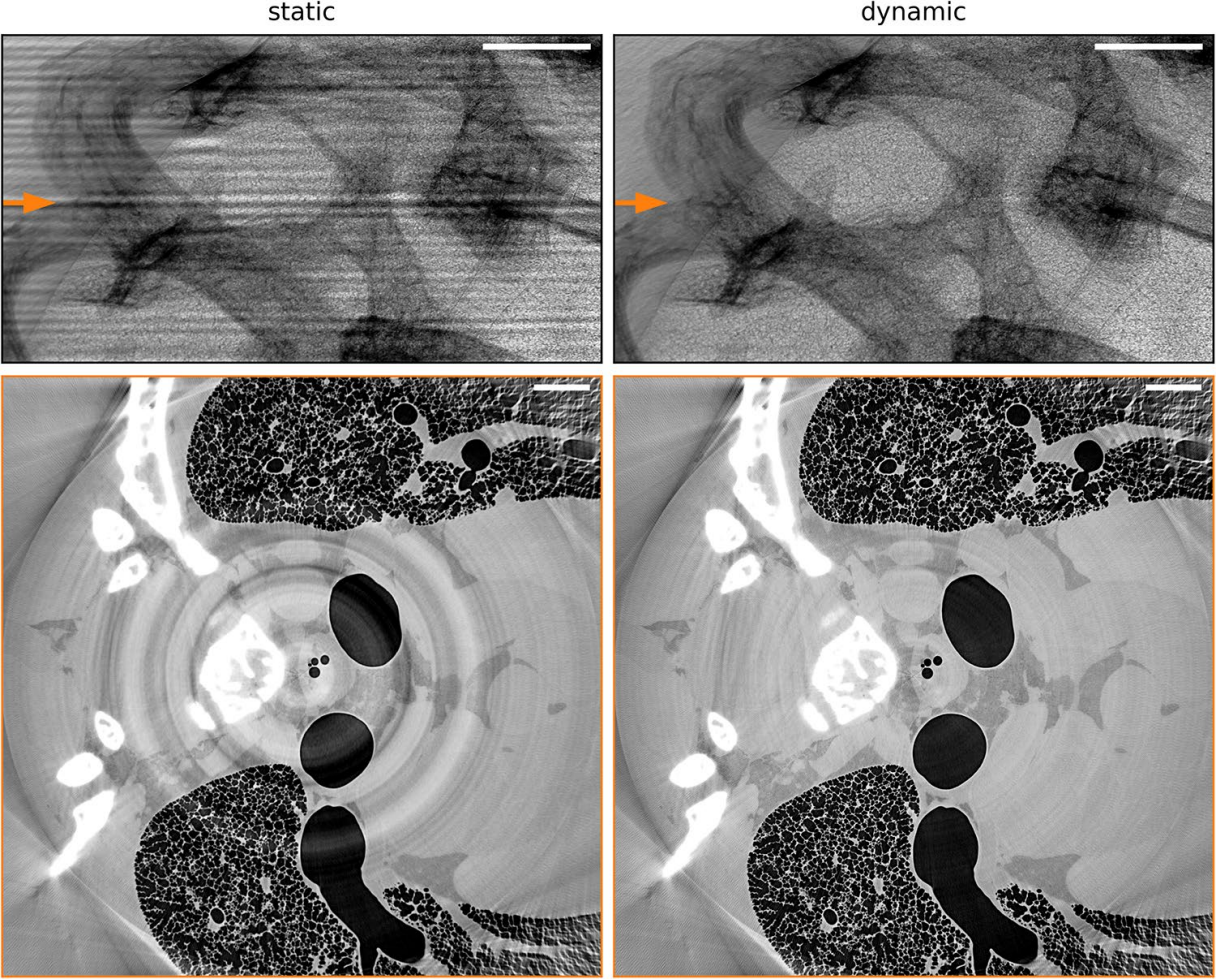
Upper panels: flat-field correction of the same projection using standard “static” and “dynamic” principal component analysis (Van Nieuwenhove et al. 2015) approaches. Lower panels: example of a reconstructed slice in a single tomographic scan, marked with the orange arrow on the upper panels, showing strong ring artefacts caused by the stripe structure (left) in the case of the “static” flat-field correction. Rings are significantly reduced when the “dynamic” flat-fielding procedure is applied (right). Scale bars: 1 mm

The process of assembling the final 3D volume from individual 360-degree tomographic scans consisted of several steps. First, all individual projections were dark-field corrected using the mean of the 50 calibration dark frames. Second, a flat-field correction was applied. Instead of a conventional (static) flat-fielding procedure (Stock 2008a), where each projection is divided by a mean/median flat-field image, a (dynamic) flat-field correction based on the principal component analysis was used (Van Nieuwenhove et al. 2015). The reason for that was a known variability of the illumination field, most probably originating from the observed vibrations of the monochromator, which becomes prominent only at short acquisitions (Ruhlandt et al. 2017). The static flat-fielding procedure by its definition relies on the stationary beam and is not designed to catch such variations. In the reconstruction process, beam fluctuations are known to contribute to a so-called fixed pattern noise and introduce ring and band artefacts (Stock 2008a; Van Nieuwenhove et al. 2015). With the dynamic approach, the ring artefacts were significantly reduced. This improvement can be seen in Fig. 2, where the same projection and an example of a reconstructed slice are compared for a static and dynamic flat-field correction.

After the dark and flat-field corrections, the projections were forwarded to a phase-retrieval process to boost the contrast between tissue and air. Phase information was extracted using a single defocused image method developed by Paganin et al. (2002). The empirical values for the δ and β parameters required by the method were set to 2.0 × 10^−7^ and 2.8 × 10^−10^, respectively, following Lovric et al. (2017a).

Finally, tomographic reconstruction from phase-retrieved projections was performed using the gridrec algorithm (Marone and Stampanoni 2012). However, the protocol used for reconstruction differed from that of Haberthür et al. (2010), where two complementary half field-of-view projections at angular positions θ and 180 + θ were stitched together and the resulting larger projection image was used as input for the reconstruction step. In the case of the present 360-degree scans, analysis of recorded projection angles revealed that the complementary projections were not separated by exactly 180°, but had a small extra 0.04565 ± 0.00007° offset. This value was larger than the angular pixel size at the edge of the detector (0.0284°). For this reason, all half-projections for each 360-degree dataset were submitted directly to the reconstruction procedure together with their known angular positions and center offsets. The small overlapping area near the rotation axis was cut off at the position of the rotation center.^1^ Alternatively, it can be also weighted with the angular positions to account for the doubling of that region (Kaestner et al. 2011). If not properly taken into account, this overlapping region would produce strong streak artefacts across the whole field of view in the reconstructions.

The rotation center was found by reconstructing a single horizontal slice using multiple rotation axis positions and choosing the one corresponding to the best reconstruction. In the present experiment, we observed a small drift of the rotation center value for scans at different vertical positions. This was due to a non-perfect parallelism of the principal rotation axis and the vertical translation stage, leading to a linear variation of the rotation axis position as a function of the vertical position. To counteract that, the rotation center was manually determined for both the top and bottom layers of the tomographic mosaic and the values for the layers in between were calculated by linear interpolation.

### Stitching of individual volumes

The mosaic of individual reconstructed volumes was assembled into a final large stack using the non-rigid stitching framework NRStitcher (Miettinen et al. 2019). As already discussed, the application of this method was essential for the stitching process, as it accounted for structural deformations in the overlapping regions of neighboring volumes, caused by a slow degradation process during the 22 min total acquisition time. Another important advantage of this method was its ability to deal with terapixel-scale data by pairwise stitching and distribution of the computational load on a computer cluster.

The final stitched 3D dataset consists of 9095 × 9106 × 7084 voxels (width × length × height) which corresponds to a physical volume of 25.0 × 25.0 × 19.5 mm^3^. Together with the full lung, it also covers chest bones and some adjacent tissue regions. The data size of the final stitched volume is about 1.2 TB. We have also extracted a sub-region from the final dataset that includes only the lung tissue. The dimensions of this sub-region are 6900 × 6900 × 6350 voxels corresponding to 18.9 × 18.9 × 17.5 mm^3^ and its dataset size is 0.6 TB.

**Fig. 3:**
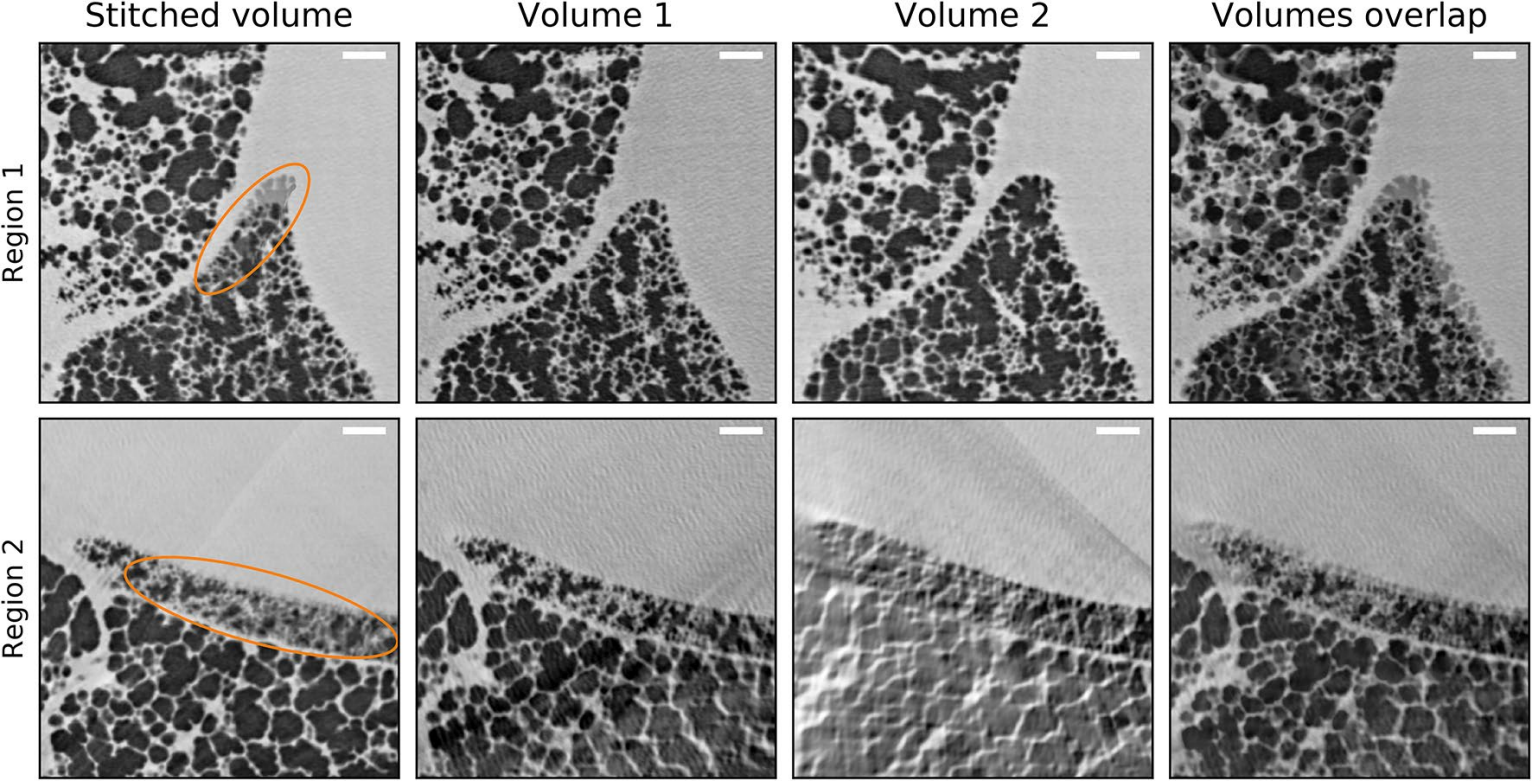
Stitching artefacts: the two horizontal rows of panels show two regions with stitching artefacts, where structures from different tomographic scans were not matched well (first column, orange ellipses). The two middle columns show two volumes with the longest separation in time before stitching. The fourth column is an overlap of the two volumes in the absence of non-rigid stitching. As discussed in the text, this mismatch is most likely caused by small regions which were not inflated at the beginning of the experiment but underwent inflation later on. Scale bars: 200 µm

The stitching procedure performed well in the vast majority of the volume, resulting in high-quality data with no apparent degradation of image resolution. During visual inspection, only two regions with stitching artefacts were discovered. Allowing larger deformations in the NRStitcher algorithm improved the output result for these regions but still could not precisely match the structures. Each region was located at the intersection of four volumes, however, the largest structural changes were observed between the two acquisitions having the longest separation in time (approximately 7.5 and 8.0 min, respectively). Figure 3 shows the affected regions in the final stitched dataset (first column) in the two single reconstructed volumes with longest time separation (middle two columns) and their overlapped view without applying the non-rigid transformation (last column). Most likely, stitching artefacts in these locations were caused by highly non-linear deformations, which the algorithm is not designed to correct. Interestingly, the two regions show much smaller structures compared to the rest of the lung volume and are located at the border of lung lobes. It is therefore possible that these regions were not fully inflated from the beginning and non-linear deformations were caused by air slowly propagating inside during the acquisition.

As an optional output, NRStitcher produces a 3D map of standard deviations of overlapping pixel values. This result can be used to identify the above-discussed regions in an automatized way and, e.g., exclude them from further analysis. Examination of the standard deviation map revealed a few more similar mismatched regions, but those exhibited far less artefacts and therefore would have been difficult to identify by visual inspection. Apart from stitching artefacts, the presence of bone tissue in the vicinity of air spaces as well as the residual ring artefacts also caused an increase in the standard deviation values. The quantitative volume fraction of all regions with artefacts had an upper limit of about 0.2% reaching a maximum of 0.9% in affected individual slices.

## Results and discussion

### Final stitched volume

**Fig. 4:**
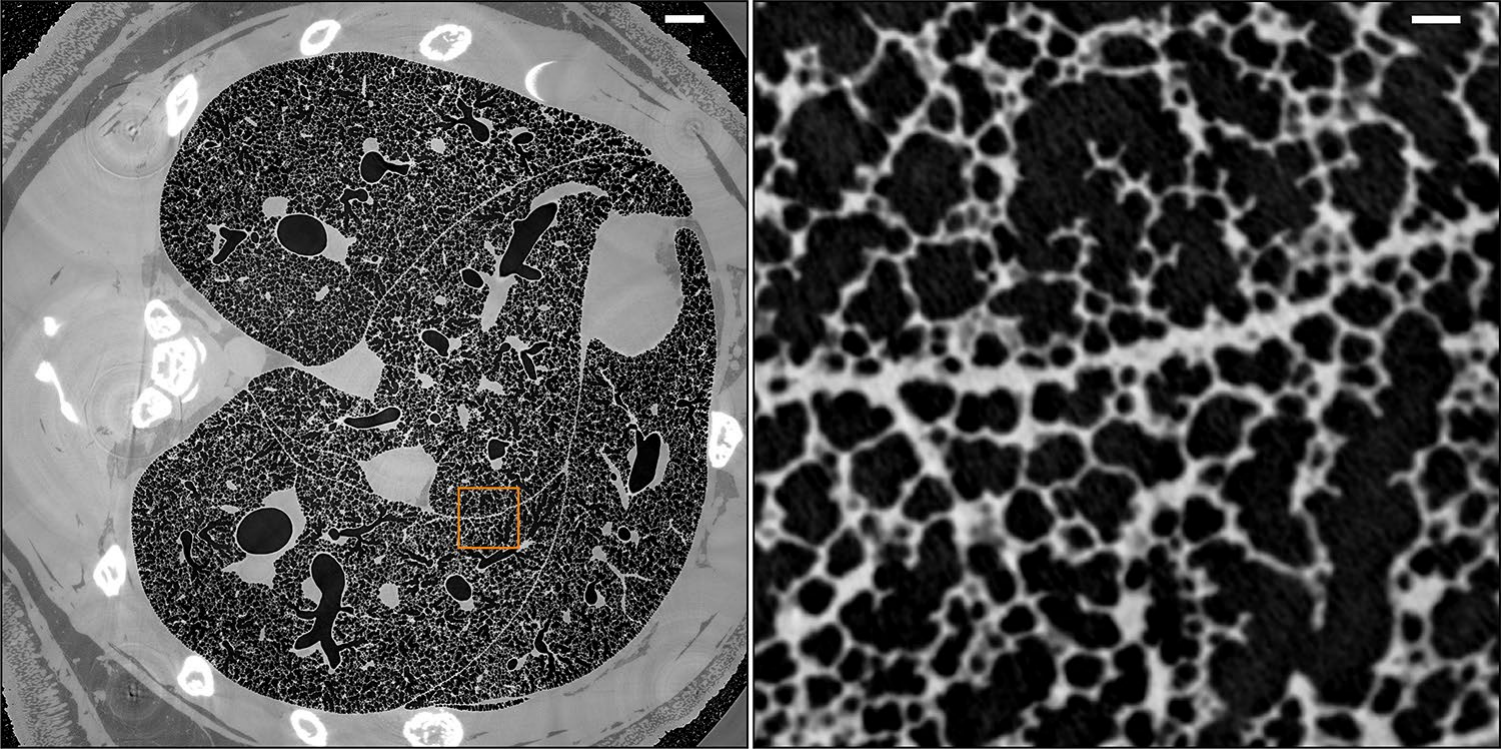
A horizontal slice through the final stitched dataset. A zoom-in into the orange square area is shown on the right panel. The full size of the area on the left is 18.9 × 18.9 mm^2^ and on the right 1.5 × 1.5 mm^2^. The long thick structure, that runs horizontally roughly in the middle of right panel, represents the border between two lobes. Even if the surfaces of the lobes move relative to each other during breathing, the capillary gap is not visible because it is filled with fluid. Scale bars: 1 mm (left panel), 0.1 mm (right panel)

A representative axial slice from the final stitched volume of the lung data is shown in Fig. 4. The left panel shows an overview over the chest region containing the lung. A closer view of the small orange square is shown on the right panel. An animated view and progressive zoom-in into this slice image can be seen in the Electronic Supplementary Material, ESM 1.

**Fig. 5:**
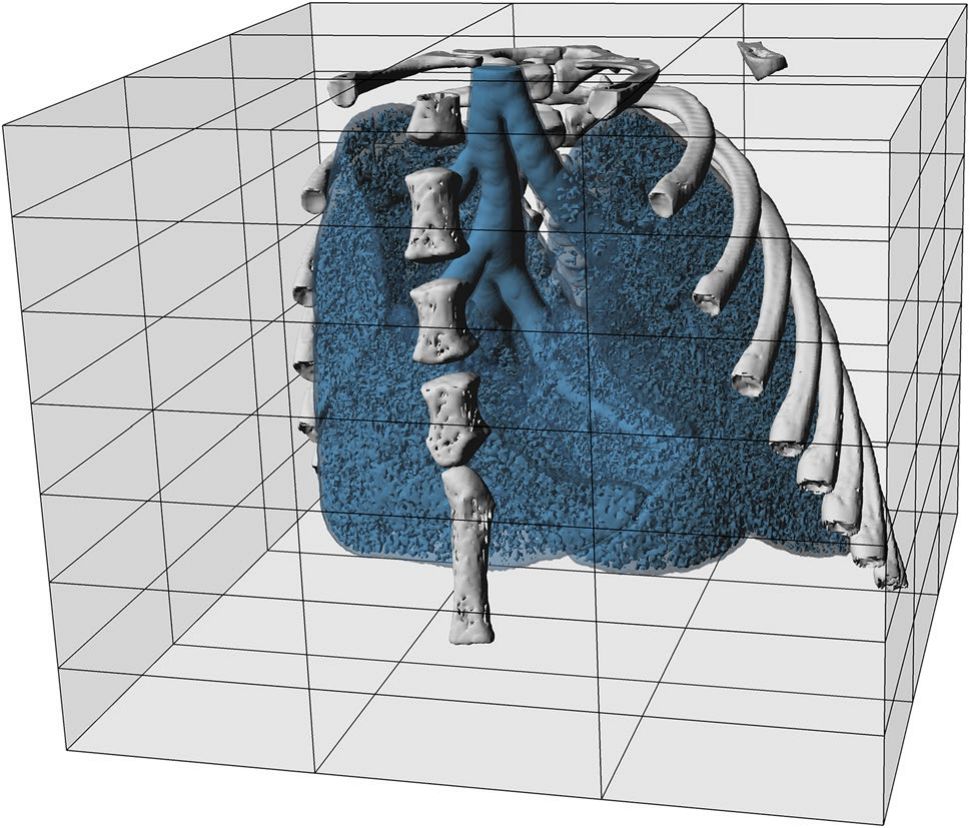
Volume view of the full data set. The pulmonary airway and air spaces of the gas exchange region (blue) are clearly visible within the animal chest. Bones are shown in grey. The cartilage connecting the ribs and the sternum is not visible because of much lower calcium content and therefore its lower contrast with the soft tissue. The semi-transparent light grey box indicates the full extent of the scanned region together with the mosaic scan pattern used to obtain the large volume (grey lines). The size of the full measured volume (light grey box) is 25.0 × 25.0 × 19.5 mm^3^ (width × depth × height)

In Fig. 5, a volume rendering^2^ of bones and air spaces is shown. The grey box marks the edges of the scanned volume with the lines indicating positions of individual wide-field scans. The same volume is rendered from different perspectives in the movie ESM 2 in the Electronic Supplementary Material.

### Segmentation

One of the first steps of analyzing and quantifying the data is segmentation. During this process, labels are assigned to parts of data corresponding to different objects, materials, etc. The segmentation process depends on the grey-level difference as well as noise levels. Thanks to the superior performance of the new optics used during the experiment (Bührer et al. 2019), the final stitched dataset shows great image quality with high contrast between the air, tissue, and bone regions (see Fig. 4), such that a simple iso-threshold technique (Velasco 1980) allows for a clear separation of the different components. Additional post-processing is still required to eliminate false detection and produce a reliable segmentation of the volume. Below, the individual steps for obtaining the segmentation of the final stitched reduced volume of the lung region are described.

- First, pixels corresponding to the bone regions were masked out. These regions showed significantly higher grey-level values in comparison with the other scan regions and were easily rejected with a single global threshold.
- Subsequently, for each slice, iso-threshold values were found for the remaining pixels to separate them into two subsets, namely air and tissue, and extract airway regions. A cylindrical mask, roughly limiting the position of the lung, was used to reject pixels at the image edges (which were located outside the animal) with grey-level values similar to those in the airway system. However, after this step the segmentation still contained non-air regions assigned to the airways and thus required further cleaning steps.
- In the next step, non-air pixels were removed from the segmentation volume by selecting only 3D connected pixels of the lung airways. The processing was done using the pi2 software package.^3^ The pi2 library distributes the image analysis tasks to a computer cluster automatically without user intervention.
- Lastly, a second iteration of the iso-threshold operation was done. The threshold values were found again for each slice using only pixels selected by a dilated segmentation from the previous step. Dilation ensured that only air and adjacent tissue pixels were used for segmentation. Additionally, a sharpness filter was applied to increase the contrast difference at the edges of the air spaces.

**Fig. 6:**
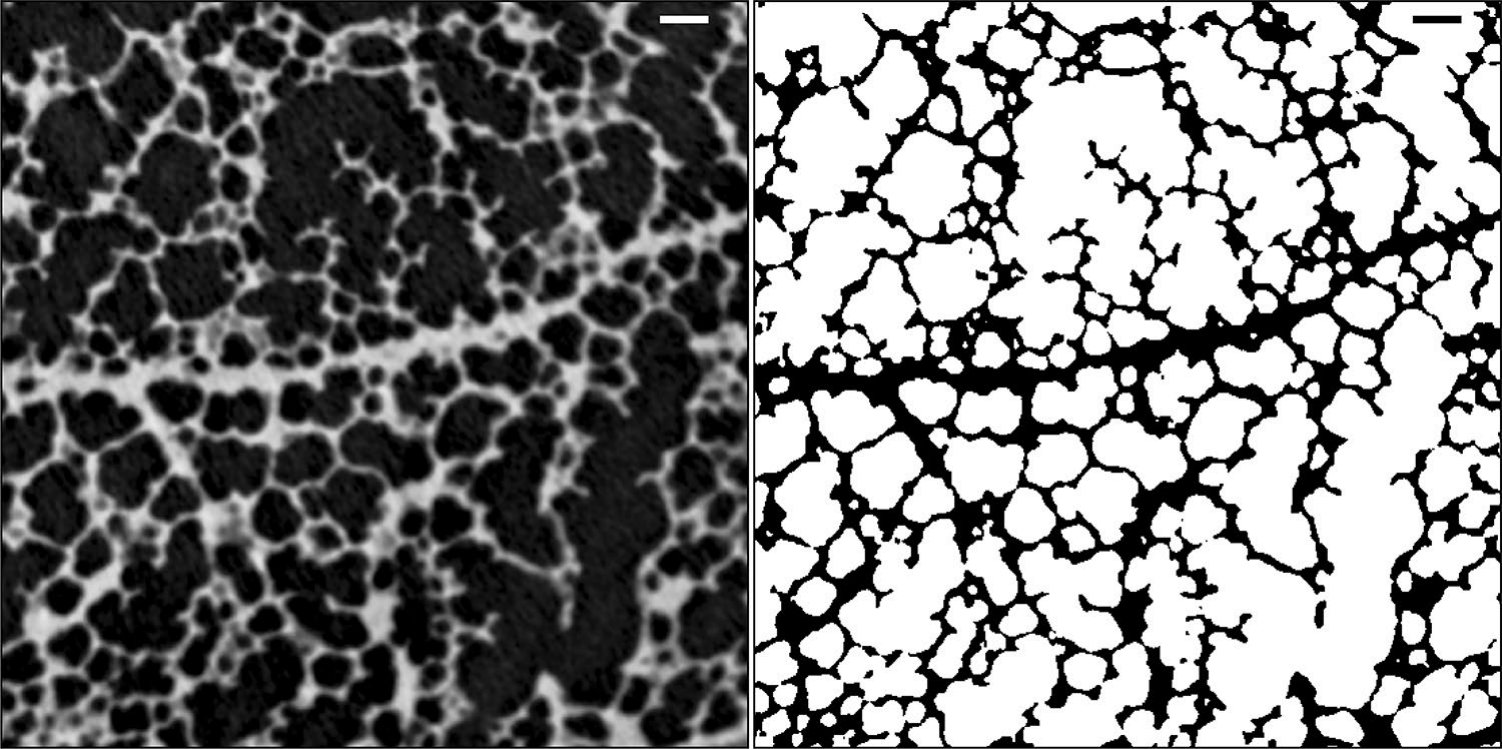
Example of the segmentation of a lung region shown in the right panel of Fig. 4. Original reconstruction (left), where airways appear black, and the segmented image (right), where the airways are marked by white color. Scale bars: left panel (white) and right panel (black):  0.1 mm

The output of the segmentation process is a binary image with the same size as the stitched image, where slices contain the label mask of the airways in the lung. An example of a segmented region is shown in Fig. 6. This mask is useful for further quantitative analysis of lung structure and morphology. A straightforward pixel count and subsequent conversion to physical units of length provides an estimate of the overall volume of the airway system of approximately 1.1 cm^3^.

In earlier studies (Lovric et al. 2017b; Oikonomidis et al. 2017a), a different and more complex approach was used to segment images with an isotropic pixel size of 1.1 × 1.1 µm^2^ to precisely identify alveolar septa. Since at the present imaging resolution, namely at pixel sizes of 2.75 × 2.75 µm^2^, alveolar septa are slightly below the resolution limit, the above described steps already yielded sufficiently good results for many quantitative analyses. However, depending on the exact nature of the analysis to be performed, more sophisticated segmentation procedures, potentially making use of prior knowledge about the sample’s structure, may have to be employed.

### Biological relevance

This work presents a micrometer-resolution tomographic dataset of a full intact juvenile rat lung. While it mainly focuses on technical details of acquisition and post-processing steps, some potential applications of this dataset for further studies are discussed in the following.

Imaging intact biological tissue is a demanding task, which puts strict technical requirements on the experimental setup. For standard X-ray tomographic imaging, various methods exist to prepare excised lungs to prevent its degradation and motion during the slow acquisition process. However, not many details are known about how much the structure of the lung is altered during the preparation process. For example, an intratracheal instillation of fixative changes the surface tension dramatically. It has to be expected that the fine structure is modified due to the alteration of the surface tension. Critical point drying causes a shrinkage of 50–60% and paraffin embedding 30–40%. Due to different physical properties of different structures, e.g. cellular versus extracellular matrix, a regular shrinkage may not be assumed. At the same token, it is not predictable if the shrinkage happens regularly or at which extent and where it is irregular. Therefore, it is important to have a high-resolution tomographic dataset of intact tissue which represents real lungs better than any dataset obtained from excised lungs.

Concerning morphological properties of the lung, still little is known about the size distribution of the pulmonary acini and alveoli. It remains a question of debate how much the volume of the acini and alveoli varies across the entire lung and if there are regions with larger or smaller variations. Further statistical analysis of the whole lung can bring a quantitative answer to this question. At the same time, it can validate the approach of previous studies of acinar properties, where only a part of the lung was scanned (Haberthür et al. 2013), by giving a number on how large this volume needs to be and which regions are the best to target. Our dataset allows such studies to be done from macro- to micro- scale on the full lung volume.

The dataset can also provide a deeper understanding of biological processes such as gas exchange and particle deposition in the lung through a quantitative characterization of the size and complexity of acini in different regions of the lung. The individual sizes and local numbers of these structural units are responsible for the absorption of particles from the air. The larger the acini are, the more efficient the deposition is. Furthermore, the deposition appears to be higher at the entrance of the acini than in the peripheral regions. From a practical point of view, this information can be used, for example, to predict the degradation of healthy lung tissue in polluted environments or to estimate the amount of medication that needs to be administered in a lung treatment. However, for both, environmental particles or drug application, the local concentrations are much more important than the average.

It is known based on studies of fixed, dried lungs that the acini in contact with the pleura (surface of the lung) are larger than all of the other acini (Kizhakke Puliyakote et al. 2016). However, even if the size of the acini is critical for ventilation and particle deposition, the size distribution of the acini in the entire lung is unknown.

Apart from morphological studies, the fast large-volume tomographic approach opens up a possibility to study, for example, the full lung development in rodents at different age stages. It has been previously shown that the sensitivity of tissue changes with age, but solid quantitative confirmation of these processes is still missing. Furthermore, this method can be used for in-detail studies of the regional microscopic effects of pharmacological and other interventions in models of lung diseases such as asthma, chronic obstructive pulmonary disease, and lung fibrosis.

The acquired dataset can be also interesting as a realistic structure animal model input for simulations to study different internal processes in the lung. The small pixel size of the dataset provides more precise surface area properties and resolves small variations in shapes, which presumably can have a large impact on, for example, gas flow.

## Conclusions

The current work presents a full high-resolution tomography mosaic scan of a complete intact juvenile rat lung recorded in situ and immediately post-mortem under slowly degrading conditions. The data acquisition technique combines a wide-field scanning method with a mosaic imaging mode and allows for maximizing the field of view during a single scan as well as avoiding motion artefacts in the reconstructed volume. The non-rigid stitching approach was used to combine the individual volumes, taking into account structural deformation occurring between individual scans. We showed that the technique has a clear advantage in imaging large non-static samples. The presented datasets (reconstructed and segmented volumes) have been published as an open access dataset (https://doi.org/10.16907/7eb141d3-11f1-47a6-9d0e-76f8832ed1b2) and may serve for future studies of lung structure and function in normal and pathological conditions at a new level of resolution and detail.

## Electronic supplementary material

Below is the link to the electronic supplementary material.

Supplementary file1 (DOCX 28 kb)

Supplementary file2 (MP4 26959 kb)

Supplementary file3 (AVI 20917 kb)

## Footnotes

1 See similar approach briefly mentioned in pyHST software package http://ftp.esrf.fr/scisoft/PYHST2/.

2 https://wci.llnl.gov/simulation/computer-codes/visit.

3 https://github.com/arttumiettinen/pi2.
