# Supplementary material for: Micrometer-resolution X-ray tomographic full-volume reconstruction of an intact post-mortem juvenile rat lung
Source: Histochem Cell Biol. 2020 Mar 18;155(2):215–26. doi: 10.1007/s00418-020-01868-8 (PMC7910225; doi:10.1007/s00418-020-01868-8)
Supplement: Supplementary file 1 — Supplementary file1 (DOCX 28 kb) [file 418_2020_1868_MOESM1_ESM.docx]

**Electronic Supplementary Materials**

**Micrometer-resolution X-ray tomographic full-volume reconstruction of an intact *post mortem* juvenile rat lung**

Elena Borisova^1,2,=^ (https://orcid.org/0000-0002-1794-3596),

Goran Lovric^1,3,=^ (https://orcid.org/0000-0002-0833-4043),

Arttu Miettinen^1,4^ (http://orcid.org/0000-0003-3132-0544),

Luca Fardin^5^ (https://orcid.org/0000-0002-9734-8825),

Sam Bayat^6,7^ (https://orcid.org/0000-0002-8565-0293),

Anders Larsson^8^,

Marco Stampanoni^1,4^ (https://orcid.org/0000-0001-7486-6681),

Johannes C. Schittny^2^ (https://orcid.org/0000-0003-4025-3961),

Christian M. Schlepütz^1,*^ (https://orcid.org/0000-0002-0485-2708)

^1^ Swiss Light Source, Paul Scherrer Institute, 5232 Villigen PSI, Switzerland

^2^ Institute of Anatomy, University of Bern, 3012 Bern, Switzerland

^3^ Center for Biomedical Imaging, École Polytechnique Fédérale de Lausanne, 1015 Lausanne, Switzerland

^4^ Institute for Biomedical Engineering, ETH Zurich, 8092 Zurich, Switzerland

^5^ European Synchrotron Radiation Facility, 38043 Grenoble, France

^6^ Department of Pulmonology and Clinical Physiology, Grenoble University Hospital, 38700 Grenoble, France

^7^ Inserm UA7, Synchrotron Radiation for Biomedicine Laboratory (STROBE), University Grenoble Alpes, 38000 Grenoble, France

^8^ Department of Surgical Sciences, Uppsala University, 75185 Uppsala, Sweden

^=^ equal contribution

^*^ Corresponding author, E-mail: [christian.schlepuetz@psi.ch](mailto:christian.schlepuetz@psi.ch)

Supplementary movie 1: ESM_1.mp4

**ESM 1** Movie showing the 3-dimensionally rendered lung volume (blue) with the surrounding bones (white) from different perspectives. Note that the dataset had to be down-sampled to be able to render it. Rendering was performed using the VisIt software (Childs et al. 2012) (<https://wci.llnl.gov/simulation/computer-codes/visit>) and the FFmpeg multimedia library (https://www.ffmpeg.org/)

Supplementary movie 2: ESM_2.avi

**ESM 2** Animation showing a continuous zoom-in into the reconstructed high-resolution slice shown in Figure 4 in the publication. The airspace of the lung structure appears in nearly black colors, while the surrounding tissue is displayed in light grey tones. The image contrast was scaled to maximize the airway-to-tissue contrast. The chest bones are saturated on this intensity scale and appear in white. Visualization was performed using the Matplotlib graphics environment (Hunter 2007) and FFmpeg multimedia library (https://www.ffmpeg.org/)

**References**

Childs H et al. (2012) VisIt: An end-user tool for visualizing and analyzing very large data. In: Bethel EW, Childs H, Hansen C (eds) High performance visualization: enabling extreme-scale scientific insight. Boca Raton, Fla. : CRC Press, Chapman & Hall/CRC computational science series, pp 357-372

Hunter JD (2007) Matplotlib: A 2D Graphics Environment. Comput Sci Eng 9:90-95 doi:10.1109/MCSE.2007.55
